# (2*E*)-3-[4-(1*H*-Benzimidazol-2-ylmeth­oxy)phen­yl]-1-(4-meth­oxy­phen­yl)prop-2-en-1-one

**DOI:** 10.1107/S1600536811012645

**Published:** 2011-04-13

**Authors:** Mehmet Akkurt, Zeliha Baktır, S. Samshuddin, B. Narayana, H. S. Yathirajan

**Affiliations:** a Department of Physics, Faculty of Sciences, Erciyes University, 38039 Kayseri, Turkey; b Department of Studies in Chemistry, Mangalore University, Mangalagangotri 574 199, India; c Department of Studies in Chemistry, University of Mysore, Manasangotri, Mysore 570 006, India

## Abstract

In the title compound, C_24_H_20_N_2_O_3_, the mean plane of the benzimidazole unit makes dihedral angles of 79.88 (11) and 85.44 (12)° with the benzene and 4-meth­oxy­benzene rings, respectively. The benzene and 4-meth­oxy­benzene rings maske a dihedral angle of 16.10 (14)°. A pair of inter­molecular N—H⋯O hydrogen bonds connects adjacent mol­ecules into an inversion dimer, generating an *R*_2_^2^(26) ring motif. The crystal structure is further stabilized by C—H⋯π inter­actions.

## Related literature

For the biological activity of benzimidazoles, see: Dhar (1981 ▶); Pujar *et al.* (1988 ▶); Bouwman *et al.* 1990 ▶); Dimmock *et al.* (1999 ▶); Satyanarayana *et al.* (2004 ▶); Madkour *et al.* (2006 ▶); Sarojini *et al.* (2006 ▶). For related structures, see: Jian *et al.* (2003 ▶); Jasinski *et al.* (2010 ▶); Odabaşoğlu *et al.* (2007 ▶). For the graph-set analysis of hydrogen bonding, see: Bernstein *et al.* (1995 ▶).
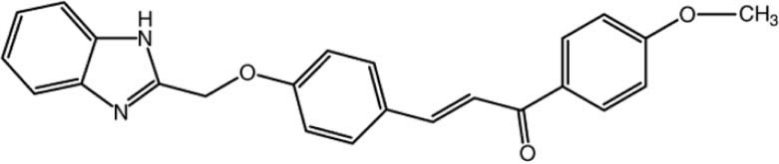


## Experimental

### 

#### Crystal data

C_24_H_20_N_2_O_3_
*M* *_r_* = 384.42
Triclinic, 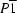

*a* = 7.2244 (5) Å
*b* = 9.3201 (8) Å
*c* = 14.9422 (9) Å
α = 98.449 (2)°
β = 99.183 (4)°
γ = 99.478 (4)°
*V* = 964.01 (12) Å^3^
*Z* = 2
Mo *K*α radiation
μ = 0.09 mm^−1^
*T* = 294 K
0.20 × 0.20 × 0.20 mm

#### Data collection

Rigaku R-AXIS RAPID-S diffractometer
Absorption correction: multi-scan (*SORTAV*; Blessing, 1995 ▶) *T*_min_ = 0.983, *T*_max_ = 0.983
20466 measured reflections
3952 independent reflections
2206 reflections with *I* > 2σ(*I*)
*R*_int_ = 0.074

#### Refinement

*R*[*F* ^2^ > 2σ(*F* ^2^)] = 0.059
*wR*(*F* ^2^) = 0.162
*S* = 1.07
3952 reflections
263 parameters
H-atom parameters constrained
Δρ_max_ = 0.19 e Å^−3^
Δρ_min_ = −0.17 e Å^−3^

### 

Data collection: *CrystalClear* (Rigaku/MSC, 2005 ▶); cell refinement: *CrystalClear*; data reduction: *CrystalClear*; program(s) used to solve structure: *SIR97* (Altomare *et al.*, 1999 ▶); program(s) used to refine structure: *SHELXL97* (Sheldrick, 2008 ▶); molecular graphics: *ORTEP-3 for Windows* (Farrugia, 1997 ▶); software used to prepare material for publication: *WinGX* (Farrugia, 1999 ▶).

## Supplementary Material

Crystal structure: contains datablocks global, I. DOI: 10.1107/S1600536811012645/xu5184sup1.cif

Structure factors: contains datablocks I. DOI: 10.1107/S1600536811012645/xu5184Isup2.hkl

Additional supplementary materials:  crystallographic information; 3D view; checkCIF report

## Figures and Tables

**Table 1:** Hydrogen-bond geometry (Å, °) *Cg*1 and *Cg*4 are the centroids of the N1/N2/C1/C6/C7 and C18–C23 rings, respectively.

*D*—H⋯*A*	*D*—H	H⋯*A*	*D*⋯*A*	*D*—H⋯*A*
N2—H*N*2⋯O2^i^	0.86	2.12	2.927 (3)	157
C11—H11⋯*Cg*1^ii^	0.93	2.58	3.490 (3)	165
C24—H24*A*⋯*Cg*4^iii^	0.96	2.61	3.467 (3)	149

Symmetry codes: (i) 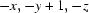; (ii) 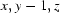; (iii) 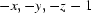.

**Fig. 1.**
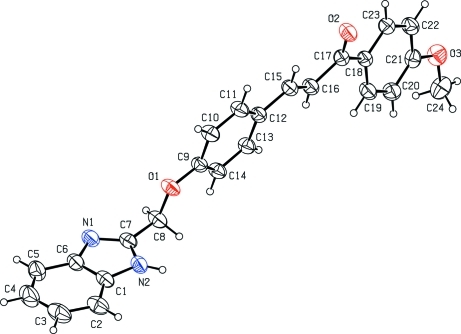
The molecular structure and numbering scheme for the title compound (I). Displacement ellipsoids for non-H atoms are drawn at the 30% probability level.

**Fig. 2.**
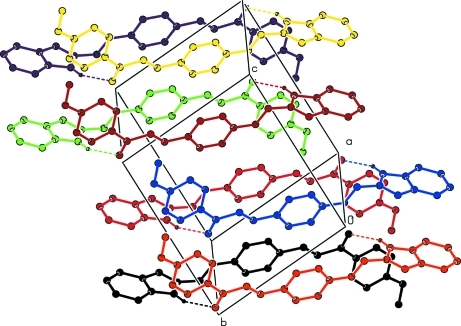
The dimer structure of (I), viewing down the a axis. Hydrogen atoms not involved in the showed interactions have been omitted for clarity.

## supplementary crystallographic information

### Comment

The benzimidazole ring system and its related compounds play an important role in pharmaceutical and agricultural fields due to their broad spectrum of biological activities (Pujar *et al.*, 1988, Bouwman *et al.*, 1990). The synthesis of novel benzimidazole derivatives remains a main focus of medicinal research. Benzimidazoles are also useful as insecticides, acaricides, nematocides, herbicides and other plant-protective agents in the field of pest control (Madkour *et al.*, 2006). In recent years, attention has increasingly been given to the synthesis of benzimidazole derivatives as a source of new antimicrobial agents. In addition, benzimidazole derivatives have played a crucial role in the theoretical development of heterocyclic chemistry and are also used extensively in organic synthesis. Chalcones constitute an important family of substances belonging to flavonoids, a large group of natural and synthetic products with interesting physicochemical properties, biological activity and structural characteristics. Chalcones are highly reactive substances of varied nature. They have been reported to possess many interesting pharmacological activities (Dhar, 1981) including anti-inflammatory, antimicrobial, antifungal, antioxidant, cytotoxic, antitumor and anticancer activities (Dimmock *et al.*, 1999; Satyanarayana *et al.*, 2004). Chalcones are also finding application as organic nonlinear optical materials (NLO) for their SHG conversion efficiency (Sarojini *et al.*, 2006).

The crystal structures of some benzimidazole derivatives *viz*., 2-chloromethyl-1*H*-benzimidazole nitrate (Jian *et al.*, 2003) and 5-methoxy-1*H*-benzo[*d*]imidazole-2(3*H*)-thione (Odabaşoğlu *et al.*, 2007) have been reported. In continuation of our work on the synthesis of benzimidazole derivatives (Jasinski *et al.*, 2010) and in view of the importance of benzimidazoles, the title compound (I) is synthesized and its crystal structure is reported here.

In the title compound (I), (Fig. 1). the dihedral angles between the least squares planes of the benzimidazole (N1/N2/C1–C7) and the benzene (C9–C14) and 4-methoxybenzene (C18–C23) rings are79.88 (11)° and 85.44 (12)°, respectively. The benzene and 4-methoxybenzene rings form a dihedral angle of 16.10 (14)° with each other.

Molecule conformation of (I) is stabilized by one weak intramolecular C15—H15···O2 interaction (Table 1). Two molecules are connected by N—H···O hydrogen bonds into an inversion dimer, forming an *R*^2^_2_(26) ring motif (Bernstein *et al.*, 1995; Table 1, Fig. 2). Furthermore, crystal packing is stabilized by C—H···π interactions (Table 1), involving N1/N2/C1/C6/C7 (centroid *Cg*1) and C18–C23 (centroid *Cg*4) rings.

### Experimental

A mixture of a 4-(1*H*-benzimidazol-2-ylmethoxy)benzaldehyde (2.52 g, 0.01 mole) and *p*-methoxy acetophenone (1.5 g, 0.01 mole) in 50 ml ethanolic sodium hydroxide was stirred at 278–283 K for 3 h, then maintained at room temperature for 24 h and poured into ice cold water. The precipitate that appeared after neutralization with dilute HCl was filtered off and recystallized from 1,4-dioxane. The single crystals were grown from DMF by slow evaporation method and yield of the compound was 75% (m.p.: 521 K).

### Refinement

H atoms were placed in geometrically idealized positions [N—H = 0.86 Å and C—H = 0.93–0.97 Å], and refined as riding with *U*_iso_(H) = 1.2*U*_eq_(C,N) for amine, methylene and aromatic H atoms or 1.5*U*_eq_(C) for methyl H atoms.

### Crystal data

**Table:**

C_24_H_20_N_2_O_3_	*Z* = 2
*M**_r_* = 384.42	*F*(000) = 404
Triclinic, *P*1	*D*_x_ = 1.324 Mg m^−^^3^
Hall symbol: -P 1	Mo *K*α radiation, λ = 0.71073 Å
*a* = 7.2244 (5) Å	Cell parameters from 1843 reflections
*b* = 9.3201 (8) Å	θ = 2.4–26.4°
*c* = 14.9422 (9) Å	µ = 0.09 mm^−^^1^
α = 98.449 (2)°	*T* = 294 K
β = 99.183 (4)°	Block, pale yellow
γ = 99.478 (4)°	0.20 × 0.20 × 0.20 mm
*V* = 964.01 (12) Å^3^	

### Data collection

**Table:**

Rigaku R-AXIS RAPID-S diffractometer	3952 independent reflections
Radiation source: Sealed Tube	2206 reflections with *I* > 2σ(*I*)
Graphite Monochromator	*R*_int_ = 0.074
Detector resolution: 10.0000 pixels mm^-1^	θ_max_ = 26.4°, θ_min_ = 2.3°
dtprofit.ref scans	*h* = −9→9
Absorption correction: multi-scan (*SORTAV*; Blessing, 1995)	*k* = −11→10
*T*_min_ = 0.983, *T*_max_ = 0.983	*l* = −18→18
20466 measured reflections	

### Refinement

**Table:**

Refinement on *F*^2^	Primary atom site location: structure-invariant direct methods
Least-squares matrix: full	Secondary atom site location: difference Fourier map
*R*[*F*^2^ > 2σ(*F*^2^)] = 0.059	Hydrogen site location: inferred from neighbouring sites
*wR*(*F*^2^) = 0.162	H-atom parameters constrained
*S* = 1.07	*w* = 1/[σ^2^(*F*_o_^2^) + (0.0423*P*)^2^ + 0.0726*P*] where *P* = (*F*_o_^2^ + 2*F*_c_^2^)/3
3952 reflections	(Δ/σ)_max_ < 0.001
263 parameters	Δρ_max_ = 0.19 e Å^−^^3^
0 restraints	Δρ_min_ = −0.17 e Å^−^^3^

### Special details

**Table:**

Geometry. Bond distances, angles *etc*. have been calculated using the rounded fractional coordinates. All su's are estimated from the variances of the (full) variance-covariance matrix. The cell e.s.d.'s are taken into account in the estimation of distances, angles and torsion angles
Refinement. Refinement on *F*^2^ for ALL reflections except those flagged by the user for potential systematic errors. Weighted *R*-factors *wR* and all goodnesses of fit *S* are based on *F*^2^, conventional *R*-factors *R* are based on *F*, with *F* set to zero for negative *F*^2^. The observed criterion of *F*^2^ > σ(*F*^2^) is used only for calculating -*R*-factor-obs *etc*. and is not relevant to the choice of reflections for refinement. *R*-factors based on *F*^2^ are statistically about twice as large as those based on *F*, and *R*-factors based on ALL data will be even larger.

### Fractional atomic coordinates and isotropic or equivalent isotropic displacement parameters (Å^2^)

**Table:**

	*x*	*y*	*z*	*U*_iso_*/*U*_eq_	
O1	0.2640 (3)	0.91719 (18)	0.15238 (11)	0.0644 (7)	
O2	0.2171 (3)	0.05475 (19)	−0.12143 (11)	0.0687 (7)	
O3	0.2682 (3)	−0.0583 (2)	−0.53944 (13)	0.0787 (8)	
N1	0.4160 (3)	1.2698 (2)	0.23251 (15)	0.0660 (8)	
N2	0.0984 (3)	1.1935 (2)	0.20220 (13)	0.0620 (8)	
C1	0.1351 (4)	1.3169 (3)	0.27147 (17)	0.0617 (10)	
C2	0.0158 (5)	1.3923 (3)	0.3165 (2)	0.0805 (14)	
C3	0.1061 (6)	1.5136 (4)	0.3826 (2)	0.0951 (16)	
C4	0.3040 (7)	1.5591 (4)	0.4022 (2)	0.1014 (16)	
C5	0.4185 (5)	1.4847 (3)	0.3566 (2)	0.0846 (14)	
C6	0.3318 (4)	1.3610 (3)	0.28934 (18)	0.0639 (10)	
C7	0.2708 (4)	1.1720 (3)	0.18325 (17)	0.0574 (9)	
C8	0.2866 (4)	1.0467 (3)	0.11237 (17)	0.0654 (10)	
C9	0.2628 (4)	0.7851 (3)	0.09807 (16)	0.0524 (8)	
C10	0.2442 (4)	0.6636 (3)	0.14123 (17)	0.0599 (10)	
C11	0.2379 (4)	0.5253 (3)	0.09186 (17)	0.0604 (10)	
C12	0.2522 (3)	0.5040 (3)	−0.00069 (16)	0.0529 (9)	
C13	0.2741 (4)	0.6283 (3)	−0.04203 (16)	0.0573 (9)	
C14	0.2791 (4)	0.7682 (3)	0.00605 (16)	0.0584 (9)	
C15	0.2423 (4)	0.3536 (3)	−0.04834 (17)	0.0594 (9)	
C16	0.2578 (4)	0.3078 (3)	−0.13370 (17)	0.0590 (9)	
C17	0.2407 (4)	0.1501 (3)	−0.17049 (17)	0.0555 (9)	
C18	0.2517 (3)	0.1048 (3)	−0.26800 (16)	0.0539 (9)	
C19	0.2228 (4)	0.1918 (3)	−0.33441 (18)	0.0656 (10)	
C20	0.2258 (4)	0.1433 (3)	−0.42634 (18)	0.0671 (11)	
C21	0.2600 (4)	0.0023 (3)	−0.45189 (18)	0.0600 (9)	
C22	0.2893 (4)	−0.0862 (3)	−0.38702 (18)	0.0640 (10)	
C23	0.2863 (4)	−0.0362 (3)	−0.29682 (18)	0.0596 (9)	
C24	0.2382 (4)	0.0284 (4)	−0.60934 (19)	0.0868 (13)	
HN2	−0.01170	1.14090	0.17640	0.0740*	
H2	−0.11640	1.36280	0.30290	0.0970*	
H3	0.03240	1.56680	0.41520	0.1140*	
H4	0.35910	1.64170	0.44720	0.1210*	
H5	0.55060	1.51550	0.36980	0.1020*	
H8A	0.18820	1.03580	0.05810	0.0780*	
H8B	0.41040	1.06430	0.09430	0.0780*	
H10	0.23600	0.67510	0.20330	0.0720*	
H11	0.22380	0.44390	0.12120	0.0730*	
H13	0.28580	0.61730	−0.10360	0.0690*	
H14	0.29320	0.84990	−0.02310	0.0700*	
H15	0.22230	0.27990	−0.01320	0.0710*	
H16	0.28010	0.37670	−0.17180	0.0710*	
H19	0.20050	0.28640	−0.31680	0.0790*	
H20	0.20550	0.20380	−0.46980	0.0800*	
H22	0.31120	−0.18090	−0.40480	0.0770*	
H23	0.30770	−0.09710	−0.25370	0.0720*	
H24A	0.11020	0.04680	−0.61620	0.1300*	
H24B	0.25670	−0.02380	−0.66660	0.1300*	
H24C	0.32740	0.12080	−0.59240	0.1300*	

### Atomic displacement parameters (Å^2^)

**Table:**

	*U*^11^	*U*^22^	*U*^33^	*U*^12^	*U*^13^	*U*^23^
O1	0.1020 (15)	0.0440 (10)	0.0521 (10)	0.0225 (9)	0.0220 (10)	0.0067 (8)
O2	0.0988 (15)	0.0499 (11)	0.0553 (11)	0.0118 (10)	0.0127 (10)	0.0072 (9)
O3	0.0908 (15)	0.0888 (15)	0.0566 (11)	0.0184 (12)	0.0189 (10)	0.0062 (11)
N1	0.0812 (16)	0.0496 (13)	0.0661 (14)	0.0156 (12)	0.0116 (12)	0.0062 (11)
N2	0.0790 (16)	0.0476 (13)	0.0561 (13)	0.0093 (11)	0.0106 (11)	0.0045 (10)
C1	0.091 (2)	0.0425 (15)	0.0544 (16)	0.0165 (14)	0.0185 (15)	0.0083 (12)
C2	0.109 (3)	0.0629 (19)	0.079 (2)	0.0315 (17)	0.0324 (18)	0.0101 (17)
C3	0.147 (4)	0.071 (2)	0.079 (2)	0.046 (2)	0.040 (2)	0.0025 (18)
C4	0.145 (4)	0.071 (2)	0.078 (2)	0.026 (2)	0.010 (2)	−0.0136 (19)
C5	0.108 (3)	0.0615 (19)	0.072 (2)	0.0123 (18)	−0.0004 (18)	−0.0038 (16)
C6	0.087 (2)	0.0465 (16)	0.0558 (16)	0.0135 (14)	0.0085 (15)	0.0064 (13)
C7	0.0799 (19)	0.0430 (15)	0.0524 (15)	0.0162 (14)	0.0154 (14)	0.0107 (12)
C8	0.098 (2)	0.0464 (16)	0.0559 (16)	0.0187 (14)	0.0212 (15)	0.0095 (13)
C9	0.0659 (16)	0.0440 (14)	0.0480 (14)	0.0162 (12)	0.0107 (12)	0.0043 (11)
C10	0.0854 (19)	0.0510 (16)	0.0483 (14)	0.0188 (14)	0.0196 (13)	0.0108 (12)
C11	0.0826 (19)	0.0463 (15)	0.0571 (16)	0.0181 (13)	0.0168 (14)	0.0140 (12)
C12	0.0614 (16)	0.0461 (15)	0.0503 (14)	0.0128 (12)	0.0089 (12)	0.0051 (12)
C13	0.0786 (18)	0.0503 (15)	0.0446 (14)	0.0173 (13)	0.0121 (12)	0.0074 (12)
C14	0.0809 (19)	0.0470 (15)	0.0504 (15)	0.0172 (13)	0.0139 (13)	0.0119 (12)
C15	0.0725 (18)	0.0461 (15)	0.0574 (16)	0.0137 (13)	0.0066 (13)	0.0062 (12)
C16	0.0737 (18)	0.0472 (15)	0.0542 (15)	0.0136 (13)	0.0088 (13)	0.0047 (12)
C17	0.0612 (16)	0.0484 (15)	0.0540 (15)	0.0106 (12)	0.0065 (12)	0.0049 (13)
C18	0.0619 (16)	0.0450 (14)	0.0518 (15)	0.0118 (12)	0.0059 (12)	0.0026 (12)
C19	0.086 (2)	0.0477 (16)	0.0610 (17)	0.0143 (14)	0.0112 (14)	0.0039 (13)
C20	0.080 (2)	0.0628 (18)	0.0590 (17)	0.0132 (15)	0.0098 (14)	0.0166 (14)
C21	0.0573 (16)	0.0678 (18)	0.0518 (15)	0.0096 (14)	0.0136 (12)	−0.0003 (14)
C22	0.0735 (19)	0.0558 (17)	0.0622 (17)	0.0178 (14)	0.0155 (14)	0.0003 (14)
C23	0.0644 (17)	0.0533 (16)	0.0598 (16)	0.0148 (13)	0.0083 (13)	0.0058 (13)
C24	0.083 (2)	0.116 (3)	0.0620 (18)	0.0126 (19)	0.0149 (16)	0.0243 (19)

### Geometric parameters (Å, °)

**Table:**

O1—C8	1.421 (3)	C17—C18	1.475 (3)
O1—C9	1.369 (3)	C18—C23	1.397 (4)
O2—C17	1.240 (3)	C18—C19	1.383 (4)
O3—C21	1.361 (3)	C19—C20	1.386 (4)
O3—C24	1.423 (4)	C20—C21	1.387 (4)
N1—C6	1.392 (4)	C21—C22	1.375 (4)
N1—C7	1.309 (3)	C22—C23	1.365 (4)
N2—C1	1.388 (3)	C2—H2	0.9300
N2—C7	1.360 (4)	C3—H3	0.9300
N2—HN2	0.8600	C4—H4	0.9300
C1—C2	1.391 (4)	C5—H5	0.9300
C1—C6	1.383 (4)	C8—H8A	0.9700
C2—C3	1.379 (4)	C8—H8B	0.9700
C3—C4	1.393 (7)	C10—H10	0.9300
C4—C5	1.364 (6)	C11—H11	0.9300
C5—C6	1.397 (4)	C13—H13	0.9300
C7—C8	1.489 (4)	C14—H14	0.9300
C9—C10	1.382 (4)	C15—H15	0.9300
C9—C14	1.387 (3)	C16—H16	0.9300
C10—C11	1.378 (4)	C19—H19	0.9300
C11—C12	1.391 (3)	C20—H20	0.9300
C12—C13	1.389 (4)	C22—H22	0.9300
C12—C15	1.461 (4)	C23—H23	0.9300
C13—C14	1.385 (4)	C24—H24A	0.9600
C15—C16	1.313 (4)	C24—H24B	0.9600
C16—C17	1.469 (4)	C24—H24C	0.9600
			
C8—O1—C9	117.93 (19)	C20—C21—C22	120.3 (2)
C21—O3—C24	117.8 (2)	C21—C22—C23	120.5 (3)
C6—N1—C7	103.6 (2)	C18—C23—C22	121.2 (2)
C1—N2—C7	106.5 (2)	C1—C2—H2	122.00
C1—N2—HN2	127.00	C3—C2—H2	122.00
C7—N2—HN2	127.00	C2—C3—H3	119.00
N2—C1—C2	132.4 (3)	C4—C3—H3	119.00
C2—C1—C6	122.9 (3)	C3—C4—H4	119.00
N2—C1—C6	104.6 (2)	C5—C4—H4	119.00
C1—C2—C3	115.8 (3)	C4—C5—H5	121.00
C2—C3—C4	122.2 (4)	C6—C5—H5	121.00
C3—C4—C5	121.2 (3)	O1—C8—H8A	110.00
C4—C5—C6	118.1 (3)	O1—C8—H8B	110.00
N1—C6—C1	111.2 (2)	C7—C8—H8A	110.00
C1—C6—C5	119.8 (3)	C7—C8—H8B	110.00
N1—C6—C5	129.0 (3)	H8A—C8—H8B	109.00
N2—C7—C8	121.4 (2)	C9—C10—H10	120.00
N1—C7—C8	124.6 (3)	C11—C10—H10	120.00
N1—C7—N2	114.0 (2)	C10—C11—H11	119.00
O1—C8—C7	107.2 (2)	C12—C11—H11	119.00
C10—C9—C14	120.1 (2)	C12—C13—H13	119.00
O1—C9—C10	115.3 (2)	C14—C13—H13	119.00
O1—C9—C14	124.6 (2)	C9—C14—H14	120.00
C9—C10—C11	119.6 (2)	C13—C14—H14	120.00
C10—C11—C12	121.8 (3)	C12—C15—H15	115.00
C11—C12—C15	118.4 (2)	C16—C15—H15	115.00
C11—C12—C13	117.4 (2)	C15—C16—H16	119.00
C13—C12—C15	124.2 (2)	C17—C16—H16	119.00
C12—C13—C14	121.8 (2)	C18—C19—H19	119.00
C9—C14—C13	119.3 (2)	C20—C19—H19	119.00
C12—C15—C16	129.1 (3)	C19—C20—H20	121.00
C15—C16—C17	121.5 (3)	C21—C20—H20	121.00
O2—C17—C18	119.5 (2)	C21—C22—H22	120.00
C16—C17—C18	119.0 (2)	C23—C22—H22	120.00
O2—C17—C16	121.5 (2)	C18—C23—H23	119.00
C19—C18—C23	117.2 (2)	C22—C23—H23	119.00
C17—C18—C19	123.9 (2)	O3—C24—H24A	109.00
C17—C18—C23	118.8 (2)	O3—C24—H24B	109.00
C18—C19—C20	122.5 (3)	O3—C24—H24C	109.00
C19—C20—C21	118.3 (2)	H24A—C24—H24B	109.00
O3—C21—C22	115.5 (2)	H24A—C24—H24C	109.00
O3—C21—C20	124.3 (2)	H24B—C24—H24C	109.00
			
C8—O1—C9—C10	178.8 (3)	C14—C9—C10—C11	−1.4 (4)
C8—O1—C9—C14	−1.1 (4)	O1—C9—C14—C13	−179.4 (3)
C9—O1—C8—C7	176.5 (2)	C9—C10—C11—C12	0.8 (4)
C24—O3—C21—C22	179.8 (3)	C10—C11—C12—C15	−179.4 (3)
C24—O3—C21—C20	−0.5 (4)	C10—C11—C12—C13	0.3 (4)
C6—N1—C7—C8	−178.8 (2)	C11—C12—C13—C14	−0.9 (4)
C6—N1—C7—N2	1.1 (3)	C13—C12—C15—C16	2.5 (5)
C7—N1—C6—C5	179.9 (3)	C15—C12—C13—C14	178.8 (3)
C7—N1—C6—C1	−1.1 (3)	C11—C12—C15—C16	−177.8 (3)
C1—N2—C7—C8	179.2 (2)	C12—C13—C14—C9	0.3 (4)
C7—N2—C1—C6	−0.1 (3)	C12—C15—C16—C17	−179.0 (3)
C1—N2—C7—N1	−0.7 (3)	C15—C16—C17—C18	177.6 (3)
C7—N2—C1—C2	178.2 (3)	C15—C16—C17—O2	−2.4 (5)
N2—C1—C2—C3	−179.5 (3)	O2—C17—C18—C19	160.7 (3)
C6—C1—C2—C3	−1.5 (4)	C16—C17—C18—C23	163.2 (3)
N2—C1—C6—N1	0.8 (3)	O2—C17—C18—C23	−16.9 (4)
C2—C1—C6—C5	1.3 (4)	C16—C17—C18—C19	−19.3 (4)
N2—C1—C6—C5	179.8 (2)	C17—C18—C23—C22	177.0 (3)
C2—C1—C6—N1	−177.7 (2)	C19—C18—C23—C22	−0.7 (4)
C1—C2—C3—C4	0.9 (5)	C17—C18—C19—C20	−177.1 (3)
C2—C3—C4—C5	−0.2 (5)	C23—C18—C19—C20	0.5 (4)
C3—C4—C5—C6	0.0 (5)	C18—C19—C20—C21	−0.3 (4)
C4—C5—C6—N1	178.3 (3)	C19—C20—C21—C22	0.2 (4)
C4—C5—C6—C1	−0.6 (4)	C19—C20—C21—O3	−179.5 (3)
N1—C7—C8—O1	102.5 (3)	O3—C21—C22—C23	179.3 (3)
N2—C7—C8—O1	−77.4 (3)	C20—C21—C22—C23	−0.4 (5)
O1—C9—C10—C11	178.8 (3)	C21—C22—C23—C18	0.7 (4)
C10—C9—C14—C13	0.8 (4)		

### Hydrogen-bond geometry (Å, °)

**Table:**

Cg1 and Cg4 are the centroids of the N1/N2/C1/C6/C7 and C18–C23 rings, respectively.

**Table:**

*D*—H···*A*	*D*—H	H···*A*	*D*···*A*	*D*—H···*A*
N2—HN2···O2^i^	0.86	2.12	2.927 (3)	157
C11—H11···Cg1^ii^	0.93	2.58	3.490 (3)	165
C24—H24A···Cg4^iii^	0.96	2.61	3.467 (3)	149

Symmetry codes: (i) −*x*, −*y*+1, −*z*; (ii) *x*, *y*−1, *z*; (iii) −*x*, −*y*, −*z*−1.
